# Ribosomal mutations enable a switch between high fitness and high stress resistance in *Listeria monocytogenes*

**DOI:** 10.3389/fmicb.2024.1355268

**Published:** 2024-03-28

**Authors:** Jeroen Koomen, Xuchuan Ma, Alberto Bombelli, Marcel H. Tempelaars, Sjef Boeren, Marcel H. Zwietering, Heidy M. W. den Besten, Tjakko Abee

**Affiliations:** ^1^Food Microbiology, Wageningen University & Research, Wageningen, Netherlands; ^2^Laboratory of Biochemistry, Wageningen University & Research, Wageningen, Netherlands

**Keywords:** pathogen, sigma B, *rpsB*, experimental evolution, food safety

## Abstract

Multiple stress resistant variants of *Listeria monocytogenes* with mutations in *rpsU* encoding ribosomal protein RpsU have previously been isolated after a single exposure to acid stress. These variants, including *L. monocytogenes* LO28 variant V14 with a complete deletion of the *rpsU* gene, showed upregulation of the general stress sigma factor Sigma B-mediated stress resistance genes and had a lower maximum specific growth rate than the LO28 WT, signifying a trade-off between stress resistance and fitness. In the current work V14 has been subjected to an experimental evolution regime, selecting for higher fitness in two parallel evolving cultures. This resulted in two evolved variants with WT-like fitness: 14EV1 and 14EV2. Comparative analysis of growth performance, acid and heat stress resistance, in combination with proteomics and RNA-sequencing, indicated that in both lines reversion to WT-like fitness also resulted in WT-like stress sensitivity, due to lack of Sigma B-activated stress defense. Notably, genotyping of 14EV1 and 14EV2 provided evidence for unique point-mutations in the ribosomal *rpsB* gene causing amino acid substitutions at the same position in RpsB, resulting in RpsB^22Arg-His^ and RpsB^22Arg-Ser^, respectively. Combined with data obtained with constructed RpsB^22Arg-His^ and RpsB^22Arg-Ser^ mutants in the V14 background, we provide evidence that loss of function of RpsU resulting in the multiple stress resistant and reduced fitness phenotype, can be reversed by single point mutations in *rpsB* leading to arginine substitutions in RpsB at position 22 into histidine or serine, resulting in a WT-like high fitness and low stress resistance phenotype. This demonstrates the impact of genetic changes in *L. monocytogenes*’ ribosomes on fitness and stress resistance.

## 1 Introduction

*Listeria monocytogenes* is a foodborne pathogen that can cause the infrequent but high-mortality disease listeriosis (Allerberger and Wagner, 2010). *L. monocytogenes* is generally considered to be a robust microorganism, capable of growing in and surviving a wide range of adverse conditions such as low pH, low temperature and low water activity (NicAogáin and O’Byrne, 2016). Microbial populations are innately heterogenous, which contributes to the spread of *L. monocytogenes* in different environmental niches, from soil to man (Abee et al., 2016; Maury et al., 2016). When a population of cells is exposed to stress, population heterogeneity can lead to the differential survival of a subset of cells, resulting in tailing of the inactivation curve.

Previously, Metselaar et al. (2015) described stress resistant *L. monocytogenes* variants, acquired after a single exposure to acid stress, with a mutation in the ribosomal *rpsU* gene, encoding small ribosomal protein S21. Additional genotypic and phenotypic studies focussed on variant V14, with a deletion that covers the entire *rpsU* gene as well as *yqeY* and half of *phoH*, and on V15 that harbors a point mutation in *rpsU* resulting in an amino acid substitution from arginine to proline in the RpsU protein, RpsU^17Arg-Pro^ (Koomen et al., 2018). Gene expression data of *L. monocytogenes* LO28 wild type (WT) and multiple-stress resistant variants V14 and V15 revealed an upregulation of 116 genes (Koomen et al., 2018), including a large fraction of genes controlled by the alternative stress sigma factor SigB, which are known to be involved in providing multiple-stress resistance (Liu et al., 2019).

In a follow-up study (Koomen et al., 2021), *L. monocytogenes* LO28 V15 was subjected, with its single RpsU^17Arg-Pro^ point mutation, to an experimental evolution protocol where variants were selected for increased fitness, defined as a higher maximum specific growth rate (μ_max_) compared to V15. Both evolved variants fixed mutations in *rpsU* (resulting in RpsU^17Pro-His^ and RpsU^17Pro-Thr^) and reverted back to WT-like high maximum specific growth rate and relative low stress resistance. The potentially disruptive effect of random insertion of a proline residue is known to alter the stability or function of proteins (Chou and Fasman, 1974). Consequently, we hypothesized that replacing the putative disruptive proline at position 17 in *L. monocytogenes* V15 with amino acids that do not have such strong disruptive effects, i.e., threonine or histidine, can restore WT-like functioning of the RpsU protein with originally an arginine at position 17. This was confirmed by using targeted mutants in *L. monocytogenes* LO28 and type strain EGDe, showing that single amino acid substitutions in RpsU enabled *L. monocytogenes* to switch between high fitness-low stress resistance and low fitness-high stress resistance.

This raised the follow-up question whether and how *L. monocytogenes* V14 could switch between low fitness-high stress resistance and high fitness-low stress resistance, since the whole *rpsU* gene is deleted and thus the known route to WT-like fitness and stress sensitivity via a single point mutation in *rpsU* is effectively blocked. Therefore, in the current study V14 was subjected to an experimental evolution regime and used a complementary genotypic, proteomic and phenotypic approach to evaluate how ribosomal mutations in *L. monocytogenes* enable a switch between fitness and stress resistance.

## 2 Materials and methods

### 2.1 Bacterial strains and culture conditions

*Listeria monocytogenes* LO28 wild type (from the strain collection of Wageningen Food & Biobased Research, The Netherlands), stress resistant ancestor V14 (Metselaar et al., 2013; Koomen et al., 2018), and evolved variants (this study) were used for all genotypic, proteomic and phenotypic analyzes. All cultures were grown as described elsewhere (Metselaar et al., 2013). In brief, cells from −80°C stocks were incubated at 30°C for 48 h on brain heart infusion (BHI, Oxoid, Hampshire), supplemented with agar (1.5% [w/w], bacteriological agar no. 1 Oxoid, Hampshire). A single colony was used for inoculation of 20 mL of BHI broth in a 100 mL Erlenmeyer flask (Fisher, United States). After overnight (ON, 18–22 h) growth at 30°C under shaking at 160 rpm, (Innova 42, New Brunswick Scientific, Edison, NJ) 0.5% (v/v) inoculum was added to fresh BHI broth. Cells were grown under constant shaking at 160 rpm in BHI at 30°C until the late-exponential growth phase (OD_600_ = 0.4–0.5).

### 2.2 Experimental evolution

Experimental evolution was performed as described in Koomen et al. (2021). Briefly, two parallel lines were inoculated with 1% (v/v) of ON culture of *L. monocytogenes* LO28 V14 in 20 mL BHI broth in 100 mL Erlenmeyer flasks. The cultures were then incubated for 24 h at 20°C with continuous shaking at 160 rpm (Innova 42, New Brunswick Scientific, Edison, NJ). For each parallel line, 44 consecutive transfers were made from 24 h-cultures, where 1% (v/v) inoculum was used to inoculate fresh BHI, resulting in about 290 generations for each of the two evolution lines (6.6 generations per culture). From every second transfer, a 700 μL culture sample was taken, mixed with glycerol (Sigma, 25% v/v final concentration), flash frozen in liquid nitrogen, and stored at −80°C, resulting in 22 stocks for both evolution lines. These stocks were revived by streaking on BHI-agar plates, from which a single colony was used to inoculate 20 mL of BHI broth in a 100 mL Erlenmeyer flask. After ON culturing at 30°C with shaking at 160 rpm, the culture was diluted 100,000 times in fresh BHI broth, and 200 μL of culture was inoculated in duplicate in wells of a honeycomb plate. The plate was incubated in a Bioscreen C (Oy growth Curves AB Ltd., Helsinki, Finland) at 30°C and the respective growth curves were determined by measuring OD_600_ over time. All growth experiments were performed with biologically independent triplicates. Stock number 14 of the first evolution line and stock number 22 of the second evolution line were streaked on BHI agar, and respective single colonies were selected to prepare −80°C stocks of 14EV1 and 14EV2.

### 2.3 Estimation of *μ*_max_

The maximum specific growth rate *μ_max_* (h^−1^) was determined at 30°C following the procedure as described previously by Biesta-Peters et al. (2010) and Koomen et al. (2021). This method is based on the time-to-detection (TTD) of five serially two-fold diluted cultures, of which the initial bacterial concentration is known. In this setup *μ_max_* equals ln(2)/generation time (i.e., *μ_max_* = 1 represents a generation (doubling) time of approximately 0.7 h or 42 min). Three biologically independent experiments were performed to estimate the mean and standard deviation of *μ_max_*.

### 2.4 Inactivation kinetics at low pH

Acid inactivation experiments were performed as described previously (Metselaar et al., 2013). Briefly, 100 mL of late-exponential phase culture was pelleted in a fixed-angle rotor (5,804 R, Eppendorf) for 5 min at 2,880 x *g*. Pellets were washed using 10 mL BHI broth and pelleted again at 5 min at 2,880 x *g*. The pellet was resuspended in 1 mL PPS, which was pre-warmed to 37°C and adjusted to pH 3.0 using 10 M of HCl, and placed in a 100 mL Erlenmeyer flask in a shaking water bath at 37°C. At appropriate time intervals, samples were taken, decimally diluted in BHI broth and plated on BHI agar using an Eddy Jet spiral plater (Eddy Jet, IUL S.A.). Plates were incubated at 30°C for 4 to 6 days for full recovery of damaged cells. Data of at least three biologically independent experiments were used for analysis.

### 2.5 Inactivation kinetics at high temperature

Heat inactivation experiments were performed as described before (Metselaar et al., 2015). Briefly, 400 μL of late-exponential phase culture was added to 40 mL of fresh BHI broth that was pre-heated to 55°C ± 0.3°C. For the determination of the initial microbial concentration, a separate Erlenmeyer with BHI at room temperature was used. Samples were taken after various timepoints and were decimally diluted in Peptone Physiological Salt (PPS). Appropriate dilutions were plated on BHI agar using an Eddy Jet spiral plater and incubated at 30°C for 4–6 days. Combined data of at least three biologically independent experiments were used for analysis.

### 2.6 Proteomic analysis

Proteomic analysis was performed on late-exponentially growing cells (OD_600_ between 0.4–0.5) of V14 and evolved variants 14EV1 and 14EV2 as described before (Koomen et al., 2021). Briefly, 2 mL of late-exponentially growing cells (OD_600_ of 0.4–0.5) cultures of the LO28 WT, V14 and evolved 14EV1 and 14EV2 were flash frozen in liquid nitrogen and stored. Samples were thawed on ice, pelleted at 17,000 x *g*, and subsequently washed twice with 100 mM Tris (pH 8). Resuspended pellets were sonicated, and samples were prepared according to the filter assisted sample preparation protocol (FASP) (Wiśniewski et al., 2009). Each prepared peptide sample was analyzed by injecting (18 μL) into a nanoLC-MS/MS (Thermo nLC1000 connected to an LTQ-Orbitrap XL) as described previously (Lu et al., 2011; Wendrich et al., 2017; Feng et al., 2022). nLC-MSMS system quality was checked with PTXQC (Bielow et al., 2016) using the MaxQuant result files.

LCMS data with all MS/MS spectra were analyzed with the MaxQuant quantitative proteomics software package (Cox et al., 2014) as described before (Smaczniak et al., 2012; Wendrich et al., 2017). Filtering and further bioinformatics and statistical analysis of the MaxQuant ProteinGroups file was performed with Perseus (Tyanova et al., 2016). Reverse hits and contaminants were filtered out. In cases where intensity values were zero, a pseudo-value of 5 was added to prevent indefinite fold changes during the t-test. Proteins were considered differentially expressed if the log_10_ transformed ratio of variant over WT [log_10_ (protein ratio)] was below −1 or above 1, with a negative log_10_ transformed Benjamini–Hochberg corrected *p*-value [−log_10_ (*p*-value)] above 2. The proteins that belonged to the SigB regulon were identified according to previous research (Kazmierczak et al., 2003; Hain et al., 2008; Ollinger et al., 2009; Oliver et al., 2010; Liu et al., 2017; Guariglia-Oropeza et al., 2018; Mattila et al., 2020). Proteins associated with the gene ontology terms “bacterial-type flagellum” or “chemotaxis” according to GOA database were identified as being linked to motility (Huntley et al., 2015). Data visualization was performed using the statistical programming language R (4.3.0).

### 2.7 RNA-sequencing

Total RNA was isolated from late-exponentially growing cells (OD_600_ between 0.4–0.5) of V14 and evolved variants 14EV1 and 14EV2. Briefly, 100 mL of late-exponential phase culture was pelleted for 1 min at room temperature (RT) at 11,000 × *g* in a fixed-angle rotor (5,804 R, Eppendorf). The pellet was resuspended in TRI-reagent (Ambion) in a beat-beater tube (lysing matrix A) by vortexing and tubes were snap frozen in liquid nitrogen until use. Cells were disrupted using a beat-beater (MP Fast Prep-24, MP Biomedicals GmbH, Eschwege, Germany) set at 6 m/s for 4 times 20 s with two minutes of intermittent air cooling per cycle. Twenty percent of the starting volume of chloroform was added, mixed and incubated at RT for 10 min. Subsequently, samples were centrifuged at 17,000 x *g* and 4°C for 15 min. The upper aqueous phase (approximately 700 μL) was transferred to an RNase free Eppendorf tube, where 600 μL of isopropanol was added, mixed and incubated at RT for 10 min. Next, the samples were centrifuged at 17,000 x *g* and 4°C for 15 min. The pellet was washed with 700 μL of ice-cold 75% ethanol, after which the pellet was centrifuged again at 17,000 x *g* for 5 min at 4°C. The pellet was resuspended in 90 μL of nuclease-free water and incubated at 60°C for 2 min to finalize RNA isolation. RNA integrity was checked using gel electrophoresis, after which the RNA was stored by adding 0.1 volume of 3 M sodium acetate at pH 5.2 with 2.5 volumes of ethanol absolute and kept at −80°C.

Before shipping the samples were centrifuges at 13,000 x *g* and 4°C for 10 min, and the supernatant was removed. The pellet was washed with 80% ethanol and centrifuged again at 13,000 x *g* and 4°C for 10 min. After removal of the supernatant and air drying, the RNA was dissolved in 90 μL of nuclease-free water and shipped on dry ice.

Ribo-Zero rRNA depletion and the generation of paired-end reads using a MiSeq system was done by BaseClear B.V. (Leiden, The Netherlands). QC and read mapping against the LO28 reference genome (NCBI accession: PRJNA664298) was performed via in-house methods by BaseClear. Counting of reads was done by htseq-count (version 0.11.1) (Anders et al., 2015). Differential expression analysis was performed using the DEseq2 package (version 1.24.0) in the statistical programming language R (version 3.6.0). Genes were considered differential expression if log_2_ (Fold Change) was below −1.58 or above 1.58, with a Benjamini–Hochberg corrected *p*-value below 0.01. The SigB regulon genes and motility related genes were annotated as described in Section 2.6.

### 2.8 SNP analysis of evolved variants

Ancestor V14 and evolved variants 14EV1 and 14EV2 obtained in the evolution experiment were sequenced using Illumina chemistry as described before (Koomen et al., 2021). Briefly, cells were pelleted and resuspended in 450 μL DNA/RNA Shield (Zymo Research) at 4°C until DNA extraction. The DNA was extracted by BaseClear (Leiden, the Netherlands) and paired-end 2 × 150 bp short-reads were generated using a Nextera XT library preparation (Illumina). A NovaSeq 6,000 system (Illumina) was used to generate paired-end reads. Raw reads were trimmed and *de novo* assembled using CLC Genomics Workbench v 10.0 (Qiagen, Hilden, Germany). SNIPPY 3.2 (Torsten, 2015), and Pilon using the “--changes” argument (Walker et al., 2014) were used for SNP analysis of evolved variants against the LO28 WT as reference.

### 2.9 Mutant construction

Mutant strains 14RpsB^22Arg-His^ and 14RpsB^22Arg-Ser^ were constructed in the V14 genetic background using the temperature sensitive suicide plasmid pAULA (Chakraborty et al., 1992). The *rpsB* gene from either variant 14EV1 or 14EV2 was amplified from genomic DNA by KAPA HiFi Hotstart ReadyMix (KAPA Biosystems, United States), using the primers listed in Supplementary Table S1. The resulting fragments were ligated in frame to the pAULA multiple cloning site via EcoR1 and Sal1 restriction that were introduced to the fragments by the respective primers. The resulting plasmid was electroporated (2.5 kV, 25 μF, 200 W), in a 0.2 cm cuvette using a BIO-RAD GenePulser, to the appropriate *L. monocytogenes* cells and plated on BHI agar at 30°C with 5 μg/mL erythromycin to select for transformants.

Two erythromycin resistant colonies per construct were inoculated in separate tubes in BHI broth supplemented with 5 μg/mL erythromycin and grown overnight at 42°C to select for plasmid integration. Selected strains resulting from a single cross-over integration event were grown overnight in BHI at 30°C to induce double crossover events and were subsequently plated on BHI agar at 30°C. Resulting colonies were replica plated on BHI with and without 5 μg/mL erythromycin and incubated at 30°C. Colonies sensitive to erythromycin were selected. PCR using the primers listed in Supplementary Table S1 and subsequent DNA sequencing of the products (BaseClear B.V. Leiden, The Netherlands) of erythromycin sensitive colonies confirmed the correct point mutation in the respective genes and the lack of additional mutations in the targeted region.

### 2.10 Statistical testing

Comparing *μ_max_* or log_10_CFU between different strains was performed in the statistical programming language R (version 3.6.0) using the t.test and var.test functions (*α* = 0.05).

## 3 Results

### 3.1 Growth kinetics of evolved variants

**Figure 1:**
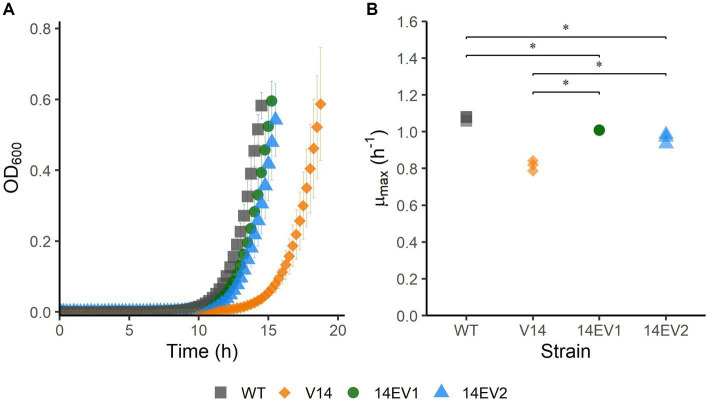
Growth performance of *L. monocytogenes* LO28 WT, V14, 14EV1, and 14EV2 at 30°C. **(A)** Growth curves for LO28 WT, V14, 14EV1, and 14EV2. **(B)** Maximum specific growth rates (μ_max_) for *L. monocytogenes* LO28 WT, V14, 14EV1, and 14EV2. The wild type is represented by squares, V14 is represented by diamonds, and variants 14EV1 and 14EV2 are represented by circles and triangles, respectively. Significant differences are indicated by an asterisk.

The experimental evolution regime was set up using two parallel cultures of *L. monocytogenes* LO28 V14. After 28 and 44 daily transfers, implicating ~186 and ~ 292 generations, respectively, this regime resulted in the selection of two evolved variants, 14EV1 and 14EV2, that showed different growth kinetics compared to the ancestor V14 (Figure 1A). The *μ*_max_ at 30°C of both evolved variants was significantly higher than that of V14, but just significantly lower than the *μ*_max_ of the original LO28 WT strain (Figure 1B). This indicated that the fitness of the evolved variants was increased compared to the ancestor V14 and almost similar to that of the WT strain.

### 3.2 Multiple-stress resistance of evolved variants

**Figure 2:**
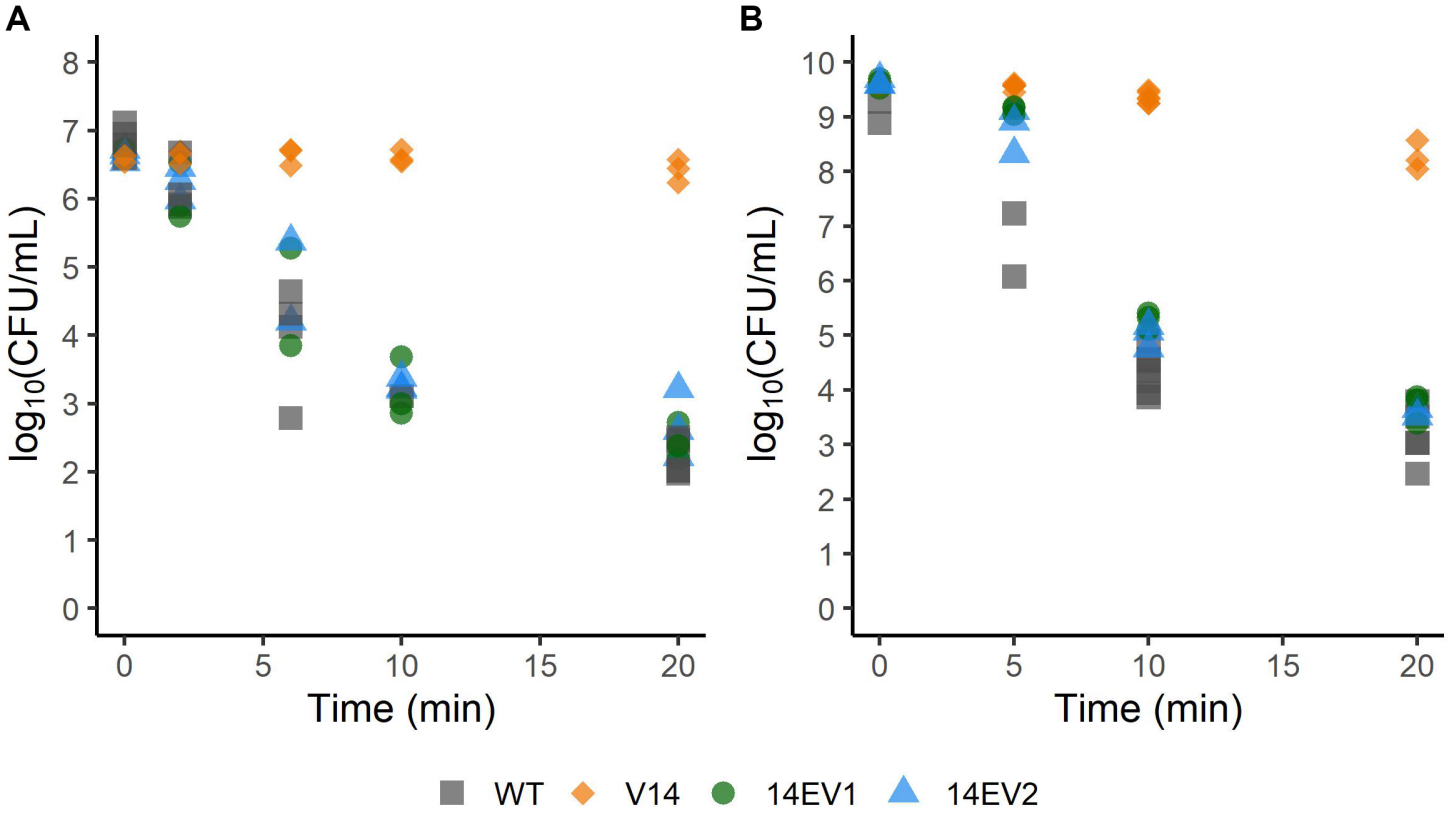
Survival of *L. monocytogenes* LO28 WT, V14, 14EV1, and 14EV2 after exposure to heat (55°C) **(A)** or acid stress (pH 3.0) **(B)**. The wild type is represented by squares, V14 is represented by diamonds, and variants 14EV1 and 14EV2 are represented by circles and triangles, respectively.

Since the evolved variants 14EV1 and 14EV2 showed increased fitness, their heat and acid stress resistance were compared to that of V14 (Figure 2). In the heat stress experiments (Figure 2A), V14 started with approximately 6.8 log_10_ CFU/mL and showed little inactivation after 20 min of exposure with a final concentration of around 6 log_10_ CFU/mL. In contrast, after 20 min of exposure the concentrations of both evolved variants 14EV1 and 14EV2 decreased and were not significantly different from the LO28 WT strain with concentrations of around 2.5 log_10_ CFU/mL. For acid stress experiments (Figure 2B), V14 again only showed a small (< 1.0 log_10_ CFU/mL) decrease in cell counts after 20 min, while both evolved variants and also the LO28 WT strain showed more than 5 log_10_CFU/mL reduction after 20 min. These data indicated that both evolved variants 14EV1 and 14EV2 lost their high resistance to heat stress and acid stress when compared to V14.

### 3.3 Proteomic and RNAseq analysis of WT and variants V14, 14EV1, and 14EV2

**Figure 3:**
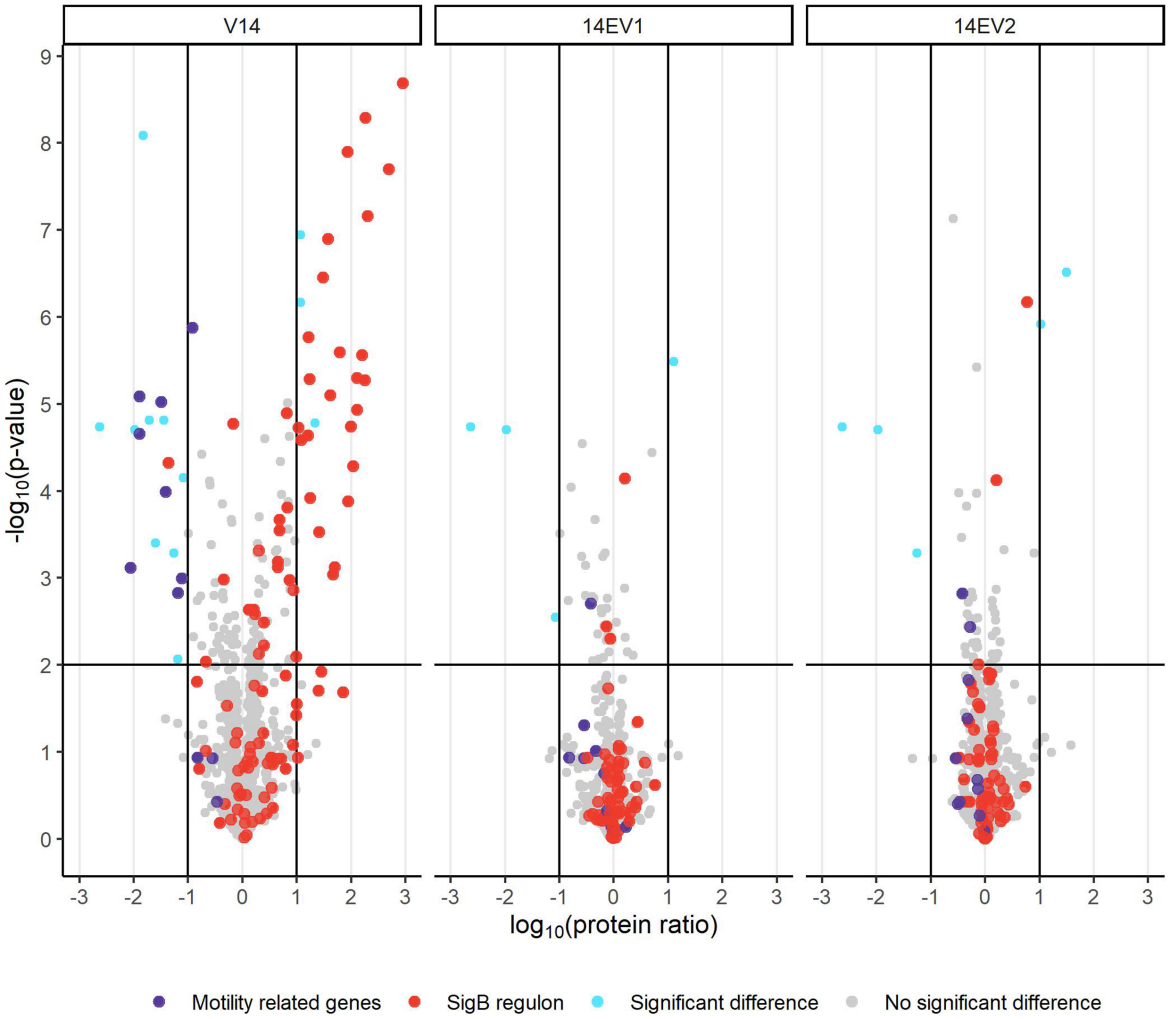
Volcano plot of proteomic data comparing *L. monocytogenes* V14, 14EV1, and 14EV2 to the wild type. The −log_10_ (*p*-value) is plotted against the log_10_ (protein ratio: variant over WT). The horizontal line represents the cutoff for −log_10_ (*p*-value), vertical lines represent log_10_ (protein ratio) cutoff. Red dots indicate proteins regulated by SigB; purple dots indicate proteins involved in motility. The expression of individual proteins is listed in Supplementary Table S2.

Comparative analysis of proteomes of late-exponential phase cells of *L. monocytogenes* LO28 WT, V14 and evolved variants 14EV1 and 14EV2 showed significant differences for V14 compared to WT and evolved variants (Figure 3; Supplementary Table S2). There were 28 proteins significantly higher expressed in V14 compared to LO28 WT, of which 25 proteins belonged to the SigB regulon (Figure 3; Supplementary Table S3). Upregulated proteins included the general stress marker Ctc (lmo0211) (Kazmierczak et al., 2003; Ferreira et al., 2004; Raengpradub et al., 2008; Oliver et al., 2010) and subunits of the known OpuC glycine betaine osmolyte transporter OpuCA (lmo1428) and OpuCC (lmo1426). SigB (lmo0895) itself was upregulated but did not pass the stringent cut-off values applied to the proteomics data (>1 or < −1 log_10_ (protein ratio), with adjusted -log_10_ (*p*-value) < 2). Comparative proteome analysis identified in total 17 proteins that were downregulated in V14 compared to the WT (Supplementary Table S3). In line with previously obtained gene expression data and the non-motile phenotype of V14 (Koomen et al., 2018), 7 of these 17 downregulated proteins are involved in motility and chemotaxis, such as MotA (lmo0685), CheA (lmo0692), and chemotaxis response regulators CheY (lmo0691) and CheV (lmo0689). Only four and five proteins were differentially expressed in 14EV1 and 14EV2 compared to the WT, respectively (Supplementary Table S3). These results indicated that in line with the return to WT-like growth kinetics of 14EV1 and 14EV2, the proteomic profiles of the two evolved variants were highly similar to that of the WT.

RNAseq data were in line with the observed results in proteomes of ancestor V14, 14EV1 and 14EV2 compared to that of the WT. In total, 281 genes were differentially expressed in V14 compared to the WT, whileas only 15 and 24 genes were differentially expressed in 14EV1 and 14EV2, respectively (Supplementary Figure S1; Supplementary Table S4). Due to the higher sensitivity of our RNAseq approach, 117 genes belonging to the SigB regulon were found as significantly upregulated in V14 when compared to the WT (Supplementary Table S5). This is in line with the 70% upregulation of the SigB regulon we reported previously based on DNA-micro array data (Koomen et al., 2018). The upregulated genes included all *opuCABCD* genes (lmo1425-1428), glutamate decarboxylase (lmo2434), and *spxA* (ArsC family transcriptional regulator, lmo2191). Other genes upregulated in the RNAseq analyzes included the virulence regulator *prfA* (lmo0200), *inlA* (lmo0433) and *inlB* (lmo0434), which encode internalin A and B involved in human epithelial cell adhesion. Genes *sigB* and *rsbX*, (serine phosphatase; indirect negative regulation of sigma B dependent gene expression) were upregulated in V14, but not in 14EV1 and 14EV2 (see Supplementary Table S6 for an overview of differential expression level of SigB regulator genes). In addition, for V14, RNAseq and proteomics analysis indicated (slight) upregulation of anti-sigma factor antagonist *rsbV* (lmo0893), anti-sigma factor *rsbW* (lmo0894) and *rsbX* (lmo0896). Notably, RsbS (lmo0890), one of the main components of the stressosome “signal integration hub” (Guerreiro et al., 2020a) was approximately 67-fold downregulated [log_10_ (protein ratio) −1.83, adjusted -log_10_ (*p*-value) > 2] in V14 compared to the WT at protein level, but the RNAseq analyzes did not show a significant difference in expression of *rsbS* between the four strains, which suggests that the observed low RsbS level in V14 is due to posttranscriptional regulation.

### 3.4 Whole genome sequencing of 14EV1 and 14EV2

Since V14 lacks the *rpsU* gene, single or multiple compensatory mutations could be expected in 14EV1 and 14EV2. Strikingly, whole genome sequencing of 14EV1 and 14EV2 revealed that both evolved lines only fixed a single nonsynonymous mutation. Both evolved variants fixed this mutation in another ribosomal protein, ribosomal protein S2 (RpsB). In the *rpsB* gene of line 14EV1, the Guanine on nucleotide position 65 mutated to Adenine [codon CGT to CAT, NC_003210.1:g.1707853G *>* A p.(Arg22His)], leading to an amino acid change from Arginine to Histidine on amino acid position 22 of RpsB (RpsB^22Arg-His^), while in 14EV2, the Cytosine on nucleotide position 64 mutated into Adenine [codon CGT to AGT, NC_003210.1:g.1707854C *>* A p.(Arg22Ser)], resulting in a substitution from Arginine to Serine on amino acid position 22 (RpsB^22Arg-Ser^). Proteomic analysis revealed no significant shifts in the levels of RpsB in V14 compared to WT, and also no significant shifts were observed in the levels of RpsB^22Arg-His^ and RpsB^22Arg-Ser^ in the evolved variants compared to the WT (Supplementary Table S2). Combining these results suggests that short term evolution experiments selecting for enhanced fitness, resulted in the isolation of 14EVs with mutations in *rpsB* to compensate for reduced fitness resulting from the loss of *rpsU*.

### 3.5 Fitness and stress resistance of constructed mutants

**Figure 4:**
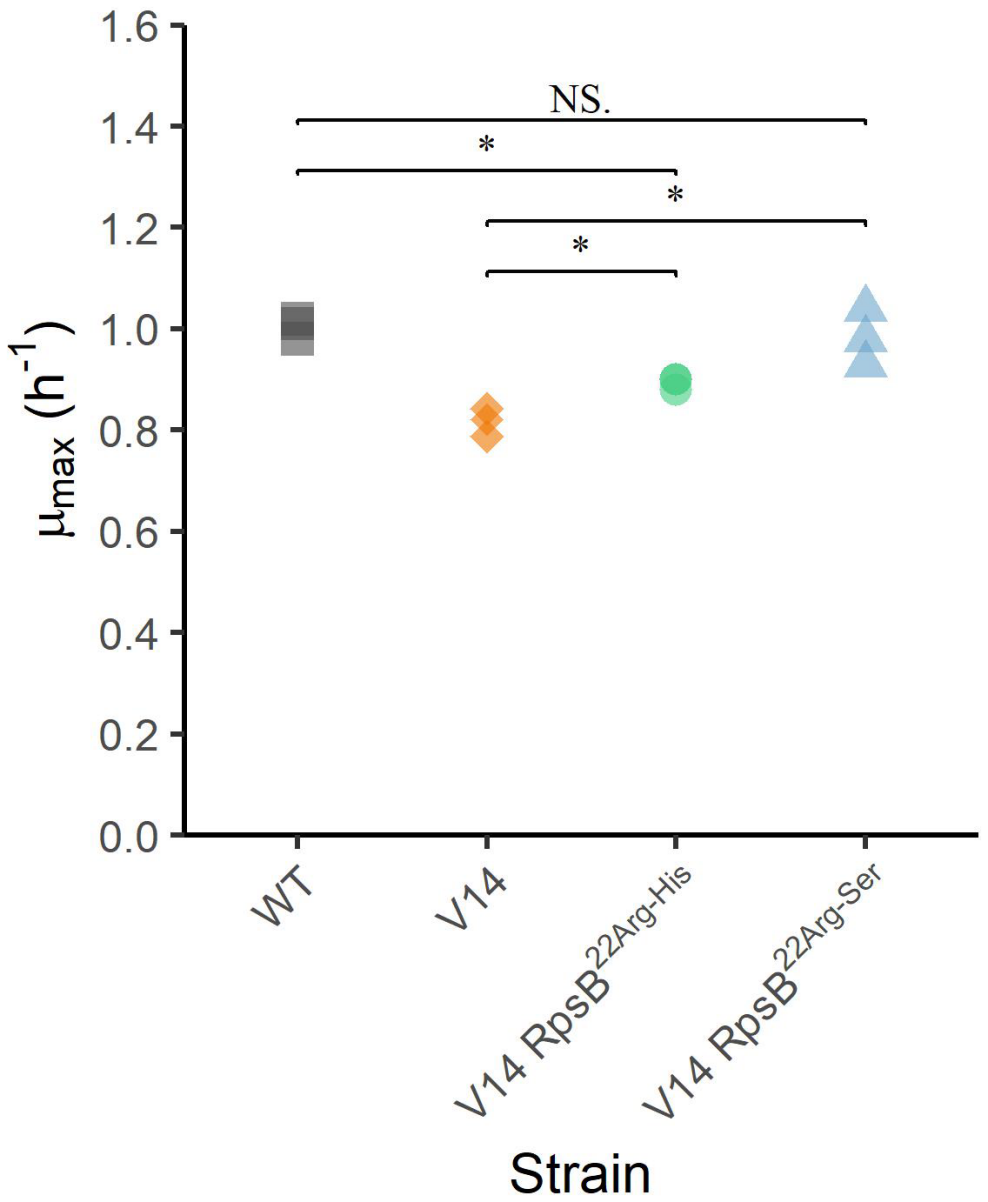
Maximum specific growth rates (μ_max_) at 30°C for *L. monocytogenes* LO28 WT, V14, and constructed mutants. Significant differences are indicated by an asterisk, and no significant differences are indicated by NS.

**Figure 5:**
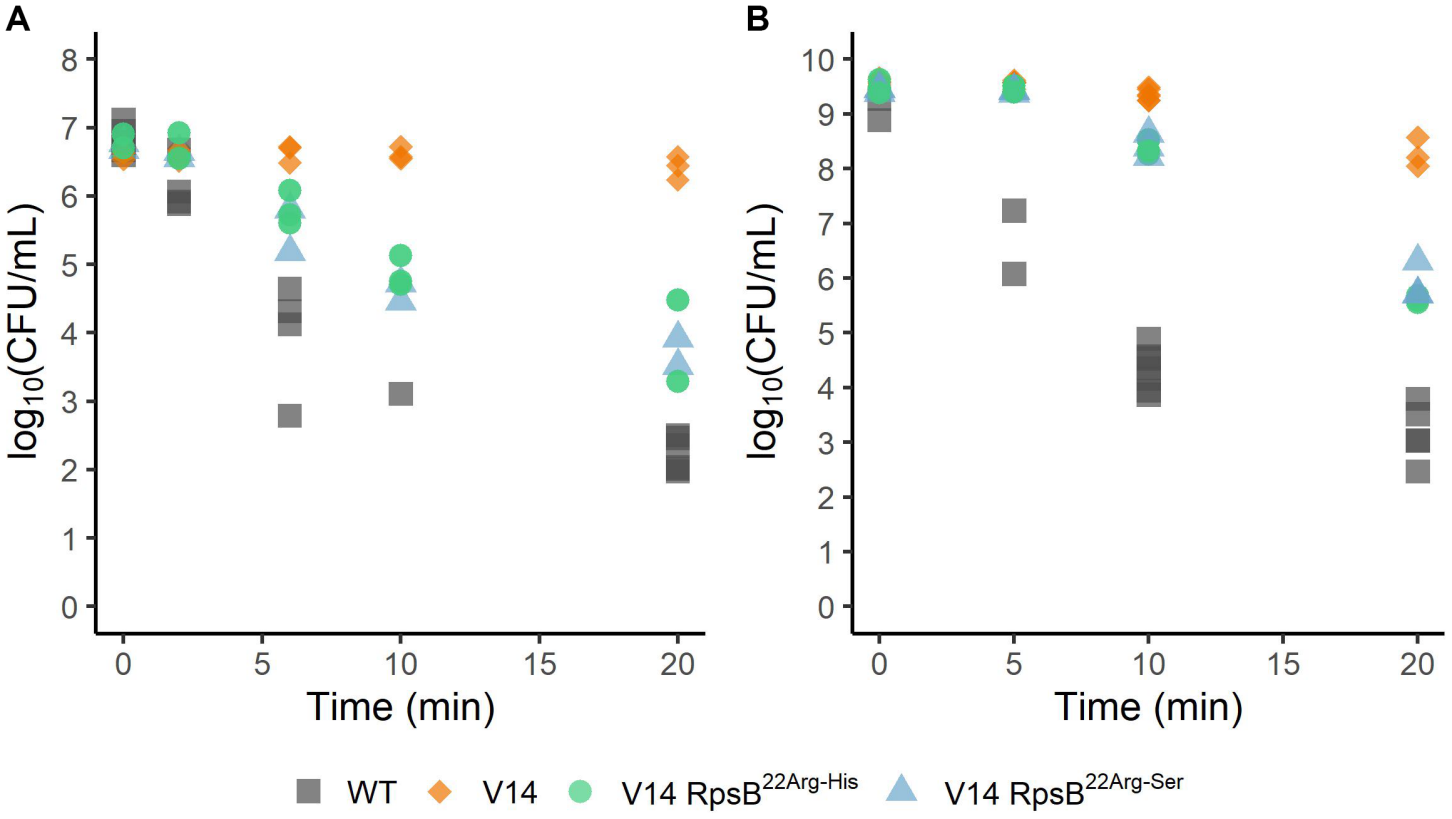
Survival of *L. monocytogenes* LO28 WT, V14, and constructed mutants, during heat (55°C) **(A)** or acid (pH 3.0) **(B)** stress. The wild type is represented by squares, V14 is represented by diamonds, and constructed mutants 14RpsB^22Arg-His^ and 14RpsB^22Arg-Ser^ are represented by circles and triangles, respectively.

To assess the effect of the substitutions that were selected during experimental evolution, RpsB^22Arg-His^ and RpsB^22Arg-Ser^ were introduced into the V14 genetic background. The *μ*_max_ was measured as a proxy for fitness and indicated that both constructed mutants of V14 had indeed a maximum specific growth rate that was significantly higher than that of V14 (Figure 4). With that of V14 carrying the RpsB^22Arg-His^ mutation significantly lower than that of LO28 WT (*p*-value = 0.001), while that of V14 carrying RpsB^22Arg-Ser^ was not significantly different from the LO28 WT (Figure 4). Subsequently, the stress response of these constructed mutants was tested, by exposure to heat (Figure 5A) and acid stress (Figure 5B). As expected, both constructed mutants were significantly less resistant to heat and acid stress after 20 min of exposure compared to V14 (*p*-value <0.05), although their resistance was still higher than LO28 WT at this timepoint.

## 4 Discussion

Previously, we described multiple stress resistance of *L. monocytogenes* LO28 variants V14 and V15 isolated after a single exposure to acid stress (Koomen et al., 2018). Stress resistance in variants V14 and V15, with a complete gene deletion or point mutation in *rpsU* respectively, was linked to induction of the SigB regulon and showed the correlation between increased stress resistance and reduced fitness. By using experimental evolution to select for increased fitness in V15 in two parallel lines, this trade-off could be revered (although not fully) via point mutations in RpsU at the same codon of the initial mutation: RpsU^17Pro-His^ and RpsU^17Pro-Thr^, respectively (Koomen et al., 2021). Here, a similar experimental evolution approach was applied using *L. monocytogenes* LO28 V14, which has a complete deletion of *rpsU*. By selecting for higher fitness in two parallel lines, two evolved variants of V14 (14EV1 and 14EV2) could be selected. Both evolved variants had higher fitness, lower stress resistance, severely reduced induction of SigB regulon members compared to V14 and a single non-synonymous mutation in the ribosomal gene *rpsB* (lmo1658). Our RNA analysis indicated that both *sigB* and *rsbX* were actively transcribed in V14.

RsbX is a SigB regulated feedback phosphatase (Xia et al., 2016) and is thought to reset the stressosome after induction, to prevent a positive feedback loop in the absence of a stress signal. In the current stressosome model (Williams et al., 2019), the phosphatase activator RsbT is released from the stressosome after phosphorylation of RsbS and acts on the signaling cascade of RsbU, RsbV, RsbW, ending in the activation of SigB. The downregulation of RsbS in V14 may have affected signaling via the stressosome. Notably, in the whole genome sequencing data of the evolved strains, no (additional) mutations that resulted in premature stop codons within the genes of the *sigB* operon were found, indicating the absence of previously described mutations in regulators of SigB expression (Guerreiro et al., 2020b). These authors showed that such mutations leading to the loss of SigB function confer a competitive advantage manifested in an increased growth rate under conditions of sublethal heat stress, at 42°C, but not in non-stressed conditions. The fact that evolved variants with higher fitness originate in our study from slow growing, multiple stress resistant V14 under non-stressed conditions, while no mutation(s) were found within genes of the SigB operon, suggests that the apparent activation of SigB regulon in V14 and loss of SigB regulon activation in 14EV1 and 14EV2, originates from alterations in ribosome functioning.

One of the stresses that can induce SigB and its operon is nutritional stress. In gram-positive bacteria, nutritional stress can indirectly affect ribosome functioning through uncharged tRNAs, leading to RelA dependent (p)ppGpp synthesis (Taylor et al., 2002; Ronneau and Hallez, 2019) In addition, nutritional stress can also lead to activation of SigB in *B. subtilis* via RelA but independent from RelA-dependent (p)ppGpp synthesis (Zhang and Haldenwang, 2003). Notably, genes involved in metabolism of branched chain amino acids (BCAA) in V14 were found to be significantly upregulated. Although *relA* (lmo1523) is not differentially expressed in our RNAseq or proteomics data, activation of the indicated pathway may point to an interplay between the SigB activation and the stringency that is affected by ribosome functioning and the mutations in the *rpsU* and *rpsB* genes. Nutritional stress-induced SigB activation has been described for *L. monocytogenes*, but how the *L. monocytogenes* stressosome and other regulator proteins respond to metabolic stress is currently unknown (Williams et al., 2019; Guerreiro et al., 2020a). The signal of energy/nutritional stress enters the SigB activation pathway probably downstream from RsbU (Shin et al., 2010). Our recent study also showed that the SigB activation in RpsU^17Arg-Pro^ mutants is independent from the stressosome and the anti-sigma factor antagonist RsbV (Ma et al., 2024). It is conceivable that altered ribosomal function in the RpsU mutant results in poor growth performance, with the mechanisms underlying SigB activation and possible link to nutritional stress requiring further study. Nevertheless, whether the ribosomal mutations lead to SigB activation via nutritional stress requires further study.

When assessing fitness and stress resistance of the constructed mutants (V14RpsB^22Arg-His^ and V14RpsB^22Arg-Ser^), WT stress sensitivity and fitness were not fully restored in the constructed mutants. While no further mutations were identified in the sequenced genome, potential influences undetectable by Illumina DNA-sequencing must be considered, such as DNA methylation, which has been known to impact translation initiation and elongation in bacteria (Wang et al., 2020), and modulation of protein activity through (de)phosphorylation reactions, particularly involving Rsb proteins that form the stressosome and regulate SigB activation (Williams et al., 2019; Guerreiro et al., 2020a).

The role of individual small (S30) and large (S50) subunit ribosomal proteins in *L. monocytogenes* has not been studied, but due to high conservation of S70 ribosome functioning, possible effects of *rpsU* and *rpsB* mutations can be discussed based on structural and functional data in well studied bacteria, including *Escherichia coli*. In *E. coli*, ribosomal protein S21 (RpsU) is part of the so-called ribosomal platform, together with S6, S11, S15, and S18 (Jagannathan and Culver, 2003; Culver and Kirthi, 2008), that functions in the initial steps of the translation process. The initiation of this translation process, the rate-limiting step for protein synthesis, depends on the assembly of translation initiation factors (IF) IF1, IF2, IF3, mRNA, and the initiator tRNA on the 30S subunit (Laursen et al., 2005; Shah et al., 2013; Keiler, 2015). This process is driven by the interaction between the mRNA’s Shine-Dalgarno (SD) sequence and the anti-SD (aSD) sequence at the 3′ end of 16S rRNA (Shine and Dalgarno, 1974; Wen et al., 2021). During the 30S subunit assembly, S2 (RpsB) and RpsU are incorporated into the 30S subunit fraction in the last stage, forming the mRNA exit channel with the 3′ end of the 16S rRNA (Sashital et al., 2014). Recent protein structure analysis of *E. coli*’s ribosomes revealed that the RpsU C-terminal residues are near the SD helix formed between the 16S rRNA aSD sequence and the mRNA SD sequence (Watson et al., 2020). Furthermore, RpsB and RpsU anchor and reinforce the binding of the ribosome and 16S rRNA to S1 (RpsA), which acts as a dynamic mesh to modulate the mRNA binding, folding and movement (Loveland and Korostelev, 2018; D’Urso et al., 2023). These findings clarify the role of RpsU during the translation initiation, including promoting base pairing between SD and aSD sequences and reinforcing RpsA’s binding to 16S rRNA. Thus, the loss or structural disruption of RpsU can divert the aSD sequence from the mRNA exit pathway, weaken RpsA binding, delay translation initiation, reduce protein synthesis, and ultimately lower growth rates. In addition, the compensatory mutations in RpsB conceivably have a positive effect on binding of RpsA to the pre-initiation complex, which enhances translation efficiency and presumably results to reversion of the trade-off between growth and stress resistance in 14EV1 and 14EV2.

**Figure 6:**
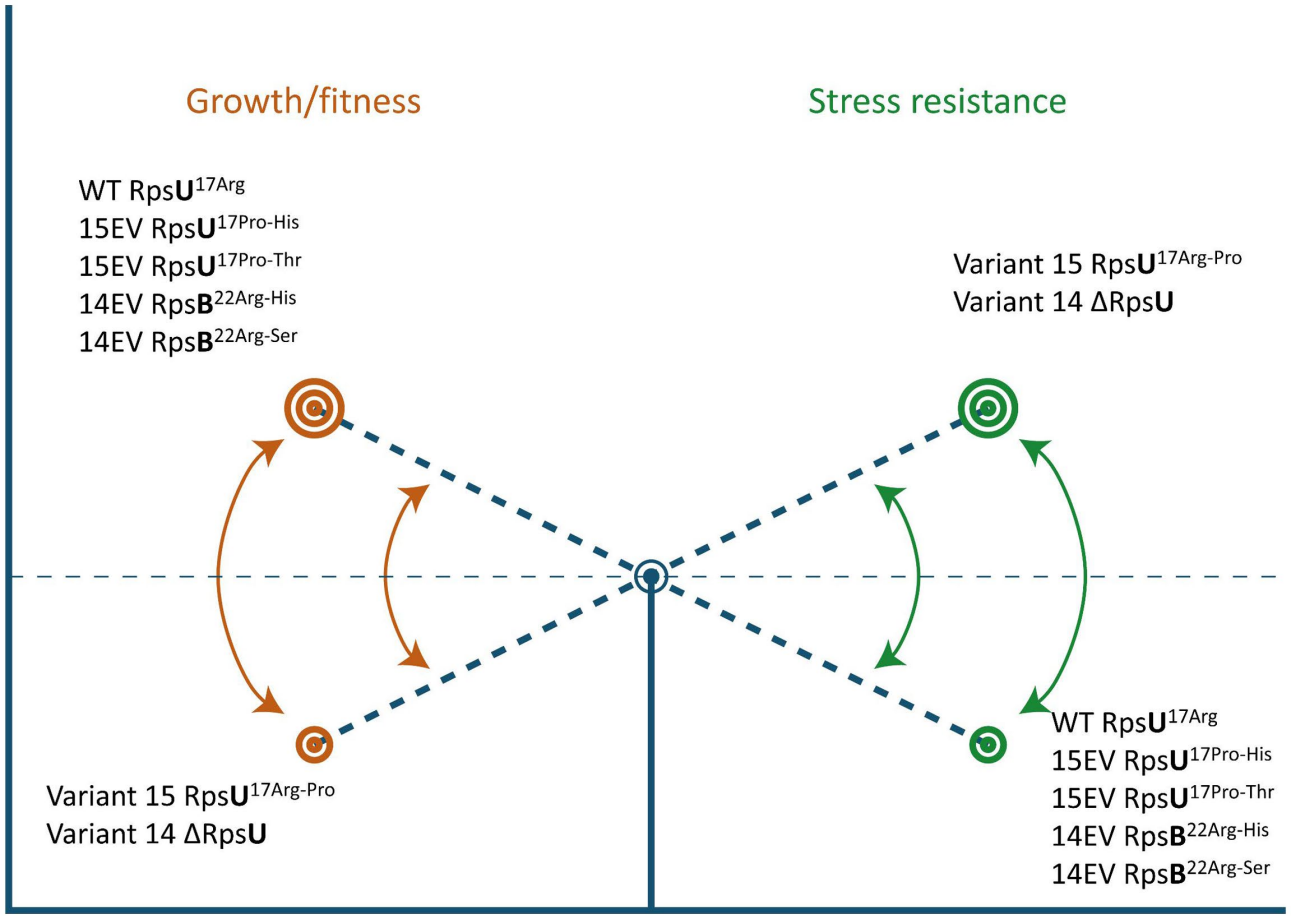
Ribosomal mutations enable a switch between high fitness and multiple-stress resistance. See text for details.

Here, we show that the apparent trade-off between increased stress resistance and lower fitness that has been described before in *L. monocytogenes* LO28 RpsU deletion mutant V14 and RpsU^17Arg-Pro^ mutant V15 (Metselaar et al., 2015; Abee et al., 2016; Koomen et al., 2018) can be reversed by compensatory mutations in *rpsB* and *rpsU*, respectively (Figure 6). Studies in yeast and higher eukaryotes have indicated that ribosomes may provide an additional layer of fine-tuning in protein expression in response to environmental factors (Gerst, 2018). However, the possibility of a dynamic ribosome, with shifts in ribosome composition and/or functionality of ribosomal proteins, via phosphorylation as a function of the environment, has mainly received attention in eukaryotes (Genuth and Barna, 2018). The results presented in the current study suggest that the 70S ribosome is involved in a signaling cascade to the SigB activation. Further work is required to elucidate in more detail the underlying mechanisms of this signaling cascade and the components involved in 70S ribosome-induced modulation of *L. monocytogenes* fitness and stress resistance.

## Data availability statement

The original contributions presented in the study are publicly available. This data can be found here: NCBI BioProject, accession PRJNA1032842; PRIDE, accession PXD022732.

## Author contributions

JK: Writing – review & editing, Writing – original draft, Visualization, Validation, Methodology, Formal analysis, Data curation. XM: Formal analysis, Writing – review & editing, Visualization, Validation, Methodology, Data curation. AB: Writing – review & editing, Validation, Data curation. MT: Writing – review & editing, Validation, Methodology. SB: Writing – review & editing, Validation, Software, Methodology, Data curation. MZ: Funding acquisition, Writing – review & editing, Validation, Supervision. HB: Funding acquisition, Data curation, Writing – review & editing, Validation, Supervision, Project administration. TA: Funding acquisition, Writing – review & editing, Validation, Supervision, Project administration.
