# Supplementary material for: Ribosomal mutations enable a switch between high fitness and high stress resistance in Listeria monocytogenes
Source: Front Microbiol. 2024 Mar 28;15:1355268. doi: 10.3389/fmicb.2024.1355268 (PMC11006974; doi:10.3389/fmicb.2024.1355268)
Supplement: Supplementary file 1 [file Table_1.DOCX]

| Supplemental Table 1: **Primers used in construction of *rpsB* mutants.**   \| Name \| Sequence (5’to 3’, restriction site underlined) \| Source \| \| --- \| --- \| --- \| \| *rpsB*-F \| TTATGAATTCTTATGACAAGAGCGAGAGCACCAA \| This study \| \| *rpsB*-R \| ACTTGTCGACTAGCGTCAGCCATTTTAGCAGTTA \| This study \| |
| --- | --- | --- | --- | --- | --- | --- | --- | --- | --- |
