# Supplementary material for: Ribosomal mutations enable a switch between high fitness and high stress resistance in Listeria monocytogenes
Source: Front Microbiol. 2024 Mar 28;15:1355268. doi: 10.3389/fmicb.2024.1355268 (PMC11006974; doi:10.3389/fmicb.2024.1355268)
Supplement: Supplementary file 7 [file Data_Sheet_1.DOCX]

| 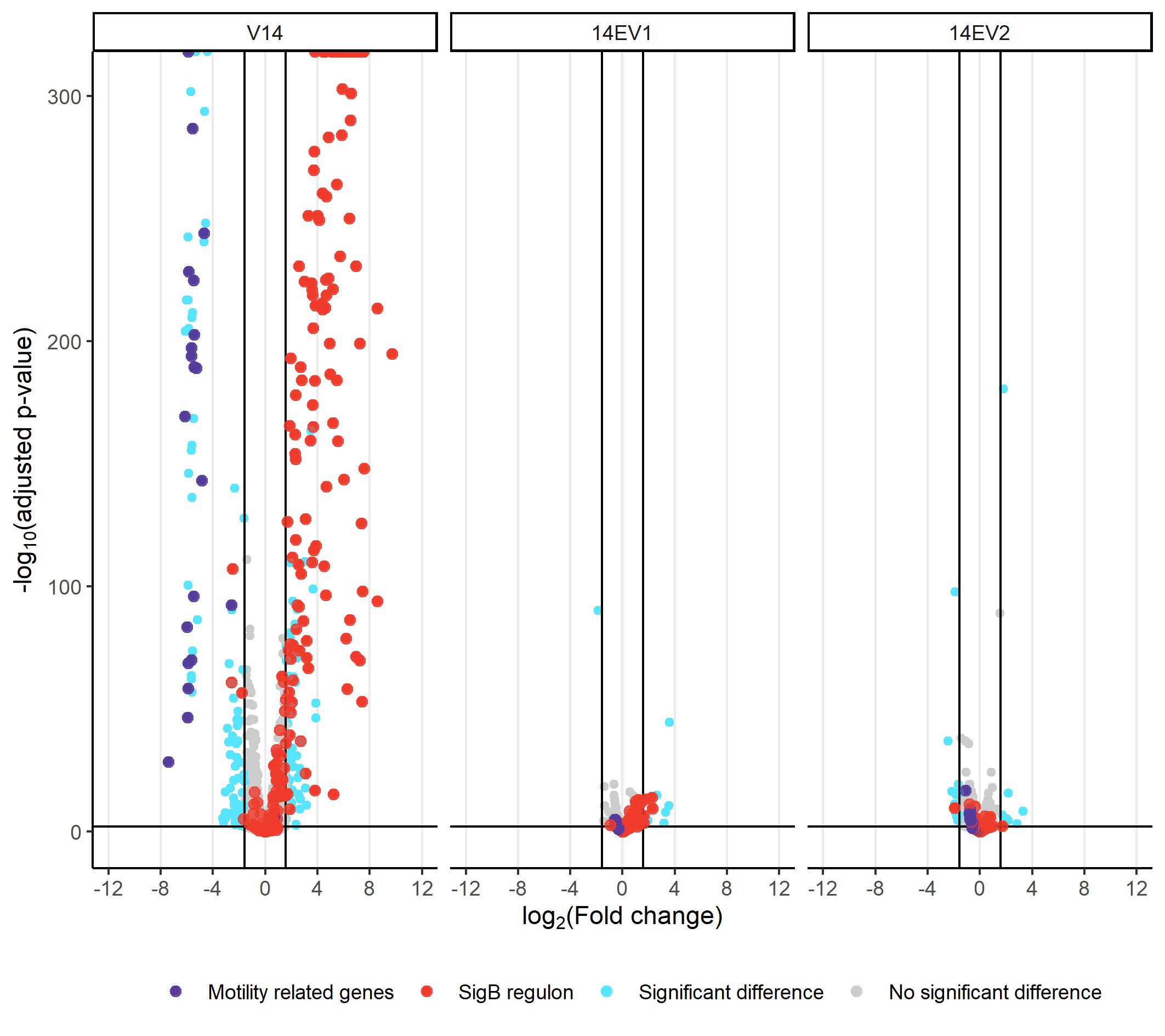  Supplemental Figure 1: **Volcano plot of RNAseq data comparing *L. monocytogenes* V14, 14EV1, and 14EV2 compared to the wild type.** The −log_10_(Benjamini–Hochberg corrected p-value) is plotted against the log_2_(Fold change: variant over WT). The horizontal line represents the cutoff for −log_10_(p-value), vertical lines represent log_2_(Fold change) cutoff. Red dots indicate proteins regulated by SigB; purple dots indicate proteins involved in motility. The expression of individual proteins is listed in [Supplemental Table 4](#tbls-RNAseq). |
| --- |
